# Reliability of ultrasound measurement of automatic activity of the abdominal muscle in participants with and without chronic low back pain

**DOI:** 10.1186/2045-709X-21-37

**Published:** 2013-11-01

**Authors:** Amir Massoud Arab, Omid Rasouli, Mohsen Amiri, Nahid Tahan

**Affiliations:** 1 Department of Physical Therapy, University of Social Welfare and Rehabilitation Sciences, Evin, Koodakyar Ave, Zip Code: 1985713831 Tehran, Iran; 2 Department of Physical Therapy, Faculty of Health Education and Social Work, Sør-Trøndelag University College (HiST), Trondheim, Norway; 3 Department of Physical Therapy, Shahid Beheshti University of Medical Sciences, Tehran, Iran

**Keywords:** Ultrasound, Reliability, Abdominal muscles, Low back pain

## Abstract

**Background:**

Ultrasound (US) imaging has been considered as a non-invasive technique to measure thickness and estimate relative abdominal muscle activity. Although some studies have assessed the reliability of US imaging, no study has assessed the reliability of US measurement of automatic activity of abdominal muscles in positions with different levels of stability in participants with chronic low back pain (cLBP). The purpose of this study was to investigate within-day and between-days reliability of US thickness measurements of automatic activity of the abdominal muscles in asymptomatic participants and within-day reliability in those with cLBP.

**Methods:**

A total of 20 participants (10 with cLBP, 10 healthy) participated in the study. The reliability of US thickness measurements at supine lying and sitting positions (sitting on a chair, sitting on a gym ball with both feet on the ground or lifting one foot off the floor) were assessed. We evaluated within-day reliability in all participants and between-days reliability in asymptomatic participants.

**Results:**

We found high ICC scores (0.85-0.95) and also small SEM and MDC scores in both groups. The reliability of the measurements was comparable between participants with and without LBP in each position but the SEMs and MDCs was slightly higher in patient group compared with healthy group. It indicates high intra-tester reliability for the US measurement of the thickness of abdominal muscles in all positions.

**Conclusion:**

US imaging can be used as a reliable method for assessment of automatic activity of abdominal muscles in positions with low levels of stability in participants with and without LBP.

## Background

Low back pain (LBP) is one of the most common musculoskeletal complaints in today’s societies, affecting up to 70-80% of the population, at least one episode during their lifetime [1]. There is a considerable evidence indicating that dysfunction of abdominal muscles is a key impairment in patients with chronic LBP (cLBP) [2-4], which might affect spinal stability [5].

The abdominal musculature are divided into the antero-lateral abdominal wall, consisting of the transversus abdominis (TrA), internal oblique (IO) and external oblique (EO); and the anterior wall, including the rectus abdominis (RA) muscle and associated fascia. The TrA muscle and posterior part of IO muscle are the part of a deep muscle cylinder that play a major role in spinal stability during functional activities [6]. The RA and EO are considered as part of a global muscle system that control spinal orientation, balance the external loads applied to the trunk and transfer load from the thorax to the pelvis [7]. Automatic activation of the abdominal muscles has been considered as a protective mechanism for lumbar spine [8]. Different patterns of abdominal muscle activation have been reported in patients with LBP compared to healthy individuals [9-11]. Ferreira et al. [9] found changes in automatic control of TrA during isometric low load tasks with the limbs suspended in people with LBP. Rasouli et al. [11] showed although the thickness change in TrA and IO increased as the stability of the position decreased in individuals with and without LBP, the amount of thickness changes were less in participants with LBP.

Assessment and measurement of the activity pattern of abdominal muscles can provide better understanding of pain behavior in patients with cLBP in the clinical environment.

A variety of measurement tools have been used by physical therapists to assess abdominal muscle activity in individuals with or without LBP. Real-time ultrasound (US) imaging has been recently used as a safe, cost-effective and feasible method to evaluate muscle structure, function and activity [12-14]. It allows real time and direct visualization and evaluation of the abdominal muscles while they contract. Changes in the thickness of abdominal muscles is measured and considered as indicator of voluntary or automatic activity of the abdominal muscles during US imaging [9,12,15]. Validity studies found good to high correlation between the muscle thickness measured by US and EMG activity in low force contractions [13,16] or those obtained using magnetic resonance imaging (MRI) [17]. McMeeken et al. [13] found good to high correlation between the needle EMG recordings of the TrA and US changes in thickness of the muscle at all activity level. Hodges et al. [16] compared the thickness changes in the abdominal muscles in real time US and EMG activity. They found that change in thickness and EMG activity of the TrA and IO muscles were linearly correlated at low contraction levels. Accordingly, using US imaging has been considered as a non-invasive technique to measure the thickness and estimate relative abdominal muscles activity.

Reliability is a psychometric property reflecting the degree to which repeated measurements provide similar results and reduction in measurement errors. Demonstrating acceptable reliability is essential for any kind of measurement and for making valid decisions. Considering lower stability, gym ball is frequently recommended in clinical practice to facilitate abdominal muscles activity. Change in the abdominal muscle thickness during sitting on a gym ball has been previously considered as automatic activity of the abdominal muscles to provide more stability [11,18]. Previous studies have investigated the reliability of US imaging in healthy individuals and patients with LBP [9,18-24] but they have mostly assessed the reliability of the antero-lateral abdominal wall muscles thickness, at rest and during voluntary abdominal muscle contraction such as abdominal hollowing manoeuvre [21,23,25-27]. The studies reported good to excellent reliability for single measures of thickness and poor to good reliability for thickness change measurements (reflecting the muscle activity). However, only a few studies evaluated the reliability of US measurements of automatic activation of abdominal muscles [9,12]. Review of the literature showed that there is no research conducted to determine the reliability of US measurement of automatic activity of abdominal muscles in sitting positions with different levels of stability. Considering the fact that gym ball is not a stable condition and during sitting on gym ball the participant’s position is instantaneously changing compared with sitting on a stable level such as a chair, abdominal muscles thickness may change in different moments and recording the US thickness measurement may be criticized.

Therefore, the purpose of this study was to evaluate within-day and between-days reliability in obtaining US thickness measurements of all abdominal muscles at supine lying and during different sitting positions (sitting on a chair, sitting on a gym ball with both feet on the ground or lifting one foot off the floor) in participants with and without cLBP.

## Methods

### Subjects

A total of 20 male volunteers (10 with chronic LBP, 10 without LBP) participated in the study. The population in this study was a sample of convenience made up of participants who were between the ages of 20 and 40 years. Patients were included if they had a history of LBP for more than six weeks before the study or had recurrent LBP and had experienced at least three episodes of LBP, each lasting more than one week, during the year before the study [28]. Asymptomatic participants were evaluated and found to have no complaint of any pain or dysfunction in their low back, pelvis, thoracic and lower extremities. Participants were excluded if they had a history of neuromuscular, musculoskeletal and cardiopulmonary diseases. The study conformed to the Declaration of Helsinki and was approved by the Human Ethics Committee at the University of Social Welfare and Rehabilitation Sciences, Tehran, Iran. All participants signed a consent form before participation in the study.

### Ultrasound measurement of the abdominal muscle thickness

A diagnostic US imaging unit set in B-mode (Ultrasonix-ES500, Canada) with a linear array transducer (7.5 MHz) was used to measure the abdominal muscles thickness in four different positions: 1) supine lying, 2) relaxed sitting comfortably on a chair 43 cm high supported against a back rest, with arms folded; hands gently resting on the opposite shoulders and the feet together on the floor, 3) sitting comfortably with a straight back on a 65 cm in diameter, gym ball with arms folded and resting on the opposite shoulders and both feet on the floor and 4) sitting on a gym ball lifting the left foot off the floor by approximately 10 cm [11,18]. Each position was held enough for the examiner to freeze a clear image of the muscle thickness at the end of expiration, usually no more than 2 minutes.

For antero-lateral abdominal wall muscles (TrA, IO, EO), the US transducer was transversely located across the right side of the abdominal wall over the anterior axillary line, midway between the 12th rib and the iliac crest, to obtain a clear image of the deep abdominal layers [13,22]. For anterior abdominal wall muscle (RA), the transducer was placed 2–3 cm above the umbilicus, 2–3 cm from the midline [22,29].

The image was frozen at the end of expiration and a vertical straight line through the center of the US image was used to ensure standardized placement of the measurement line. The cursor points carefully measured the muscle thickness between the inside edge of fascial bands in millimeter (mm). The US transducer was not displaced during the testing procedure.

### Procedures

Within-day intra-tester reliability of the US thickness measurements of the abdominal muscles was assessed in all participants (with and without cLBP) while between-day reliability was investigated in asymptomatic participants. To establish within-day reliability, at first the examiner performed measurements in all tested positions in participants and then after 60 minutes repeated the measurements in a blinded fashion and in a random order with the same procedure. The participants testing positions and the order of measurements were randomly selected to reduce the memory effect. For between-days reliability, the US measurements in all positions were repeated after one week in asymptomatic participants (N = 10). All testing procedure was performed in the biomechanics laboratory of the Department of Physical Therapy in the University of Social Welfare and Rehabilitation Sciences, Tehran, Iran.

### Data analysis

Kolmogrov-Smirnov test was utilized to assess the normality of distribution for US measurement of muscle thickness at different testing positions. Normal distribution was observed for variables.

Two measurement sessions were implemented in one day for within-day reliability in all participants. Third session of measurement was done one week later in asymptomatic participants for between-days reliability assessment. The intra-class correlation coefficient (ICC) has become the preferred index for test-retest reliability as it reflects both correlation and agreement [30]. The ICC, two way mixed effect model, was used to assess intra-tester reliability of the measurements. We calculated the ICC (3,1) as described by Shrout and Fleiss [31] because only one judge evaluated the same population.

Relative reliability values such as ICC are not sufficient to interpret in the context of an individual score. Therefore, we calculated standard error of measurement (SEM) and minimal detectable change (MDC) to make a judgment about the degree that measurements vary for an individual. The SEM is useful in reliability studies to determine the range of scores that would be expected from one assessment session to the next. The SEM was calculated using the formula: [SEM = SD √1-r] where SD is the standard deviation calculated from the measurements and r is the reliability coefficient for that measurement, in this case, the calculated ICC (3,1) value and, then, the coefficient of variation was calculated (CV in %: SEM/mean value for the given parameter × 100) [21].

Power analysis was used to determine the sample size for test. Type I error (α) was set at 0.05 and power of the test was 0.80. Considering this, it seems that calculated sample size in this study was appropriate to test the hypothesis and the results derived from the study are meaningful.

## Results

The demographic data for both LBP and healthy control groups are summarized in Table 1.

**Table 1:** Demographic data of the participants in each group (Mean ± SD)

**Variables**	**Without LBP (n = 10)**	**With LBP (n = 10)**
Age (years)	25 ± 3	26 ± 3
Weight (kg)	75 ± 7	76 ± 8
Height (m)	1.75 ± 0.06	1.75 ± 0.06
BMI (kg/m^2^)	24.3 ± 0.9	24.6 ± 0.7

*SD* Standard deviation, *LBP* Low back pain, *BMI* Body mass index.

Table 2 shows the US measurements of the absolute abdominal muscle thicknesses (Mean ± SD) in mm in two groups for different assessments during different testing positions. The smallest absolute abdominal thicknesses were related to supine lying position and the highest values belonged to position 4 when participant sat on the gym ball and lifted his left foot off the floor in both healthy and cLBP groups. The absolute thickness of TrA ranged from 3.5 ± 0.5 mm to 6.6 ± 0.8 during four positions in healthy group and from 3.5 ± 0.8 to 4.9 ± 1 mm in LBP group. The absolute thicknesses of IO,EO and RA ranged from 9.2 ± 1.4 to 12.8 ± 2.1; 5.8 ± 1.1 to 7.5 ± 0.9 and 12.9 ± 2 to 16.9 ± 2.2 mm during mentioned positions in healthy participants respectively, and from 8.3 ± 1.1 to 10.5 ± 2.5; 5.8 ± 1.1 to 7.9 ± 2.6 and 14 ± 3.7 to 17.5 ± 5.4 mm respectively in patients with LBP (Table 2).

**Table 2:** The (Mean ± SD) scores for the ultrasound thickness measurements (in mm) of the abdominal muscles during different testing positions in both groups

**Position**	**Muscle**	**Without LBP**			**With LBP**	
		**First assessment**	**Second assessment**	**Third assessment**	**First assessment**	**Second assessment**
P 1	TrA	3.6 ± 0.6	3.5 ± 0.5	3.8 ± 0.6	3.5 ± 0.8	3.7 ± 1.0
	IO	9.2 ± 1.4	9.3 ± 1.3	9.2 ± 1.5	8.4 ± 1.5	8.3 ± 1.1
	EO	5.8 ± 1.1	5.9 ± 1.2	5.9 ± 1.0	5.8 ± 1.1	6.1 ± 1.4
	RA	12.9 ± 2.0	13.0 ± 2.3	13.1 ± 2.1	14.0 ± 3.7	14.2 ± 4.3
P 2	TrA	4.0 ± 0.7	3.9 ± 0.8	3.9 ± 0.9	3.6 ± 0.8	3.6 ± 0.8
	IO	9.4 ± 1.5	9.4 ± 1.3	9.5 ± 1.8	8.1 ± 1.2	8.3 ± 1.3
	EO	6.2 ± 1.2	6.0 ± 1.1	5.9 ± 1.2	6.4 ± 2.0	6.7 ± 2.0
	RA	13.1 ± 2.0	13.2 ± 2.1	13.4 ± 2.0	15.8 ± 5.2	15.3 ± 4.8
P 3	TrA	4.4 ± 0.7	4.5 ± 0.6	4.4 ± 0.5	3.8 ± 0.9	4.0 ± 0.7
	IO	10.0 ± 1.6	10.0 ± 1.7	10.3 ± 1.6	8.8 ± 2.2	9.1 ± 2.3
	EO	6.2 ± 1.1	6.54 ± 1.3	6.3 ± 1.1	6.6 ± 2.2	6.9 ± 2.6
	RA	13.9 ± 2.1	14.1 ± 2.4	14.2 ± 2.1	16.2 ± 5.4	16.4 ± 6.0
P 4	TrA	6.6 ± 0.8	6.3 ± 0.6	6.5 ± 0.8	4.9 ± 1.0	4.1 ± 1.0
	IO	12.8 ± 2.1	12.8 ± 2.1	12.6 ± 2.0	10.5 ± 2.5	10.5 ± 2.3
	EO	7.5 ± 0.9	7.4 ± 1.2	7.4 ± 1.0	7.9 ± 2.6	7.9 ± 2.2
	RA	16.5 ± 2.5	16.8 ± 2.8	16.9 ± 2.2	17.2 ± 4.8	17.5 ± 5.4

*SD* Standard deviation, *LBP* Low back pain, *TrA* Transversus abdominis, *IO* Internal oblique, *EO* External oblique, *RA* Rectus abdominis, *P 1* Supine lying, *P 2* Sitting on chair with both feet on the ground, *P 3* Sitting on gym ball with both feet on the ground, *P 4* Sitting on gym ball lifting the left foot off the floor.

Table 3 presents the ICC (3,1), SEM, CV (SEM as % mean) and MDC values for within-day and between-days reliability of US thickness measurements of abdominal muscles were taken during different testing positions in each group. Depending on the muscle (TrA, IO, EO and RA) and participant’s position (supine lying or various sitting positions), reliability of thickness measurements ranged from 0.88 to 0.95 for within-day comparisons and from 0.85 to 0.94 for between-days comparisons in healthy group and from 0.89 to 0.94 for within-day comparisons in LBP group which indicated high intra-tester reliability for the US measurement of thickness of abdominal muscles in all positions (Table 3).

**Table 3:** ICC, SEM and MDC, CV values for within-day and between-days reliability of the ultrasound measurements at different testing positions

**Position**	**Muscle**	**Without LBP**								**With LBP**			
		**Within-day**				**Between-days**				**Within-day**			
		**(Between-trial)**								**(Between-trial)**			
		**ICC**	**SEM**	**MDC**	**CV%**	**ICC**	**SEM**	**MDC**	**CV%**	**ICC**	**SEM**	**MDC**	**CV%**
P 1	TrA	0.92	0.19	0.52	5.4	0.90	0.21	0.58	5.8	0.91	0.25	0.69	6.9
	IO	0.92	0.20	0.55	2.1	0.92	0.20	0.55	2.1	0.92	0.26	0.71	3
	EO	0.90	0.23	0.63	3.9	0.88	0.26	0.71	4.5	0.89	0.34	0.93	5.7
	RA	0.88	0.28	0.77	2.1	0.85	0.31	0.85	2.4	0.93	0.25	0.69	1.7
P 2	TrA	0.95	0.30	0.82	7.6	0.92	0.38	1.05	9.7	0.94	0.37	1.02	10.2
	IO	0.93	0.40	1.10	4.5	0.93	0.40	1.10	4.2	0.93	0.39	1.07	4.8
	EO	0.94	0.38	1.05	6.1	0.91	0.47	1.29	9	0.94	0.53	1.46	8.1
	RA	0.93	0.52	1.43	3.9	0.93	0.52	1.43	3.9	0.94	0.56	1.54	3.6
P 3	TrA	0.90	0.34	0.93	7.7	0.86	0.40	1.10	9	0.90	0.39	1.07	10
	IO	0.92	0.31	0.85	3.1	0.90	0.34	0.93	3.4	0.93	0.49	1.35	5.3
	EO	0.92	0.29	0.80	4.6	0.89	0.33	0.91	5.3	0.92	0.56	1.54	8.3
	RA	0.93	0.22	0.60	1.6	0.87	0.31	0.85	2.2	0.93	0.65	1.79	4
P 4	TrA	0.94	0.40	1.10	6.3	0.89	0.54	1.49	8.3	0.93	0.78	2.15	17.3
	IO	0.94	0.41	1.13	3.2	0.94	0.41	1.13	3.2	0.92	1.27	3.50	12
	EO	0.92	0.52	1.43	7	0.93	0.48	1.32	6.4	0.92	1.23	3.39	15
	RA	0.94	0.55	1.51	3.3	0.90	0.71	1.96	4.3	0.94	0.93	2.57	5.3

*SD* Standard deviation, *ICC* Intra-class correlation coefficient, *SEM* Standard error of measurement, *MDC* Minimal detectable change, *CV* Coefficient of variation, *LBP* Low back pain, *TrA* Transversus abdominis, *IO* Internal oblique, *EO* External oblique, *RA* Rectus abdominis, *P 1* Supine lying, *P 2* Sitting on chair with both feet on the ground, *P 3* Sitting on gym ball with both feet on the ground, *P 4* Sitting on gym ball lifting the left foot off the floor. All SEM and MDC values are in millimeter.

Generally we had higher SEM in position 4 than position 1 in both groups and SEMs of patient group (0.25 to 1.27 mm) were rather higher than corresponding SEMs of control group (0.19 to 0.71 mm), and, when expressed as a percentage (CV) of the corresponding mean thicknesses ranged from 1.6 to 9.7% (in healthy participants) and 1.7 to 17.3% (in LBP patients) (Table 3).

Likewise SEM trends in two groups, MDCs were higher in position 4 than supine lying in both groups and MDCs of patient group ranged from 0.69 to 3.50 mm and were slightly higher than corresponding MDCs of control group (0.52 to 1.96 mm) (Table 3).

## Discussion

Physical therapists have recently utilized US imaging to evaluate muscle function, structure and activity and change in the thickness of abdominal muscles is measured and considered as indicator of voluntary or automatic activity of the abdominal muscles in this method [12,14,15]. However, like any other measurements demonstrating adequate reliability is a prerequisite for using US measurement as a valid measure of abdominal muscles activity to make decision especially in clinical setting. According to Domholdt [32], the reliability is not a fixed property but it depends on the studied population and testing positions.

To our knowledge, this is the first study to collectively evaluated within-day and between-days reliability of US measurement of automatic activity of abdominal muscles in sitting positions with different stability levels in healthy participants and within-day reliability in patients with cLBP.

The results of this study suggest that there is not only high reliability of muscle thickness in asymptomatic participants but also in patients with cLBP and the results also are consistent with the available literature on the topic, finding high ICC scores and also very small SEM and MDC scores [21,23].

In the patient group, the ICCs were almost the same as the control group during each testing position and it is important for using US to evaluate abdominal muscle activity in patients with LBP in the clinical setting. The ICCs of thickness measurements ranged from 0.85 to 0.95 for within-day and between-days comparisons depending on the muscle and participant’s position which indicated high intra-tester reliability for the US measurement of abdominal muscle thickness in all positions. Norasteh et al. [22] reported good reliability (ICC: 0.81- 0.97) of abdominal muscles (TrA, IO, EO, RA) assessment by US imaging in asymptomatic participants and patients with acute LBP. In another study, Costa et al. [12] reported high reliability (ICC: 0.75- 0.94) for absolute thickness and moderate reliability (ICC: 0.26- 0.72) for thickness changes in US measurements of automatic activity of TrA, IO and EO muscles in patients with cLBP. However, in this study we assessed the reliability of absolute muscles thickness measurements and thickness changes were not analyzed.

In practical terms, the SEM shows more useful information than the ICC [33] and the CV is very good to compare the relative measurement error of variables with differing absolute values or measurement units. In the current study, the SEMs in position 4 were higher than position 1 in both groups and the SEMs of patient group (0.25- 1.27 mm) were rather higher than corresponding SEMs of control group (0.19- 0.71 mm), and, the CVs of the corresponding mean thicknesses ranged from 1.6 to 9.7% (healthy participants) and from 1.7 to 17.3% (cLBP patients). The SEMs were consistent with prior error measurements from other accepted techniques like Teyhen et al. [23] found the SEM of 0.13 to 0.31 mm for the TrA muscle. The CV in this study was similar to the results of previous studies which reported 11–14% for the reliability of TrA thickness and whole abdominal mass thickness [21,23]. All the SEMs and the corresponding CVs were low, so make them promising measures for further studies.

The MDC is especially useful for interpreting the relevance of any changes recorded after an intervention [34]. The MDCs of abdominal muscles were higher in position 4 than supine lying in both groups, the same as SEM values and MDCs of patient group ranged from 0.7 to 3.5 mm and were slightly higher than corresponding MDCs of 0.5 to 1.9 mm in control group. Critchley et al. [35] reported MDC of 0.6 (TrA) and 1.2 (OI) mm in supine position, and Bunce et al. [36] found values ranging from 0.9 to 1.8 mm for TA only in supine, sitting and standing. The differences between our results with previous studies are related to the positions and level of contractions for example if we just exclude the values of position 4, we have a range of 0.5 to 1.8 mm. which are similar to those recorded at rest and hence appear to be acceptable from the perspective of human performance measurements [37].

Reliability can be influenced by several factors such as the participants, examiner and clinical or experimental setting. In addition, there are some sources of error which might affect procedure of US measurement including accuracy of marking the fascial bands, position of patient/transducer and detection of landmarks [12].

The high reliability of our US measurements may be due to the fact that the clear image was frozen on the screen and the muscle thickness between the fascial bands was measured carefully by cursor points. Furthermore, the US transducer was not displaced during the testing procedure as much as possible and the positions were the same for every participant.

This study was not investigated the issue of validity for US imaging. Considering the results of this study and validity studies [16,17], US imaging can be used to assess and compare the automatic activity of the abdominal muscles in positions with different stability level between participants with and without cLBP.

## Conclusion

In agreement with previous studies, the results of this study demonstrate high within-day and between-days reliability for US thickness measurements of the abdominal muscles at supine lying and sitting position with different levels of stability in healthy participants and high within-day reliability in patients with cLBP.

These results indicate that real-time US imaging can be reliably used to evaluate and compare the automatic activity of the abdominal muscles between participants with and without cLBP. This might also help to determine the efficacy of prescribed treatments for cLBP patients.

### Limitation

This study assessed the reliability of US thickness measurements of the abdominal muscles in men. The results might not be extrapolated to the women. More studies are needed to collectively perform this study in men and women. The other point was that the study was only limited to a younger age group.

Another area of concern is that the between-day reliability was assessed in the asymptomatic participants. For between-days reliability, the US measurements were repeated after one week. It was possible that the pain intensity changed after one week in LBP patients. Change in pain intensity could affect the trunk stabilizer muscles such as abdominal muscles. Since the change in pain could not be completely controlled after one week, we assessed between-day reliability in asymptomatic participants with no LBP. This issue can be considered as a limitation in this study.

It is suggested to assess the within-day and between-day reliability for automatic activation of the abdominal muscles during different tasks in the patients with cLBP.

### Clinical implication

US imaging can be used as a reliable method to evaluate and compare the automatic activity of abdominal muscles in different positions with different levels of stability in participants with and without cLBP and this might help to determine the efficacy of prescribed treatments for cLBP patients.

### Ethical approval

This research was reviewed and was approved by the Human Subject Committee at University of Social Welfare and Rehabilitation Sciences.

## Competing interests

AMA contributed to conception, design, analysis, interpretation of data and drafting the manuscript. OR carried out the data collection and drafting the manuscript. MA participated in design and interpretation of data. NT participated in data collection and helped to draft the manuscript. All authors read and approved the final manuscript.

## Authors’ contributions

The authors declare that they have no competing interests.
